# Lipid Disturbances in Psoriasis: An Update

**DOI:** 10.1155/2010/535612

**Published:** 2010-07-20

**Authors:** Aldona Pietrzak, Anna Michalak-Stoma, Grażyna Chodorowska, Jacek C. Szepietowski

**Affiliations:** ^1^Department of Dermatology, Venereology and Paediatric Dermatology, Medical University of Lublin, ul. Radziwillowska 13, 20-080 Lublin, Poland; ^2^Department of Dermatology, Venereology and Allergology, Wrocław Medical University and Ludwik Hirszfeld Institute of Immunology and Experimental Therapy, Polish Academy of Sciences, ul. R. Weigla 12, 53-114 Wrocław, Poland

## Abstract

Psoriasis is a common disease with the population prevalence ranging from 2% to 3%. Its prevalence in the population is affected by genetic, environmental, viral, infectious, immunological, biochemical, endocrinological, and psychological factors, as well as alcohol and drug abuse. In the recent years, psoriasis has been recognised as a systemic disease associated with numerous multiorgan abnormalities and complications. Dyslipidemia is one of comorbidities in psoriatic patients. Lipid metabolism studies in psoriasis have been started at the beginning of the 20th century and are concentrated on skin surface lipids, stratum corneum lipids and epidermal phospholipids, serum lipids, dermal low-density lipoproteins in the psoriatic skin, lipid metabolism, oxidative stress and correlations between inflammatory parameters, lipid parameters and clinical symptoms of the disease. On the basis of the literature data, psoriasis can be described as an immunometabolic disease.

## 1. Introduction

Psoriasis is a common disease affecting, as presumed, approximately 120–180 million people worldwide [1]. Around 150,000 new cases of psoriasis are reported annually. There are fewer reports on the incidence of psoriasis, but in recent studies an increasing trend over the last 3 decades was shown [1, 2]. The population prevalence of psoriasis has been reported to range from 2% to 3%. However, in some countries there is a higher prevalence rate for psoriasis, for example in Kazakhstan, Trinidad and Tobago, Paraguay, Kenya, Tanzania, Egypt, and Kuwait [3]. Four hundred people die annually from psoriasis-related causes in the Unites States [1]. Psoriasis prevalence in the population is affected by genetic, environmental, viral, infectious, immunological, biochemical, endocrinological, and psychological (trauma, stress) factors as well as alcohol and drug abuse [4, 5].

In the recent years, psoriasis has been recognised as a systemic disease associated with numerous multiorgan abnormalities and complications. In psoriatic patients an increased risk of cardiovascular abnormalities, hypertension, dyslipidemia, atherosclerosis, diabetes mellitus type 2, obesity, chronic obturative pulmonary disease, cerebral stroke, osteoporosis, cancer, and depression was noticed [6–8].

**Figure 1:**
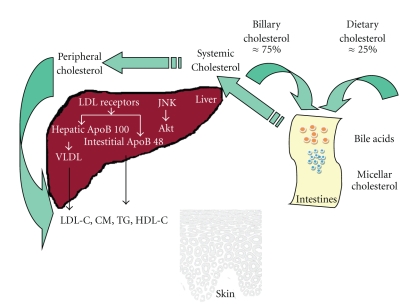
Cholesterol trafficking in human organism. CM: chylomicrones, HDL-C: high-density lipoproteins cholesterol, LDL-C: low-density lipoproteins cholesterol, VLDL-C: very low-density lipoproteins cholesterol, TG: triglycerides, JNK: Janus-family tyrosine kinase, and Akt: kinase Akt. The figure is adapted after permission from [13]. The complete electronic version of this article can be found online at: http://www.lipidworld.com/content/8/1/41.

Lipid metabolism research studies in psoriasis have been started at the beginning of the 20th century from the quantitative analysis of serum cholesterol in psoriatic patients [9]. The abnormal fat metabolism was considered to be an important factor in the etiopathogenesis of psoriasis. Grütz and Burger examined the development of psoriatic skin manifestations as a symptom comparable to xanthomatosis [9, 10]. Melczer found changes in the composition of phospholipids in psoriatic foci and suggested that inflammation, congestion, and parakeratosis resulted from lipid deposition in the reticular-endothelial system [11]. It was also suggested that continuous separation of psoriatic scales caused the permanent loss of lipids which might affect serum lipid abnormalities [11, 12]. Lipid metabolism is a complex process which takes place in different human organs and peripheral blood (Figure 1) [13]. Its disturbances in psoriasis need further studies to be fully elucidated. There are some new methods for diagnosis of cholesterol in the healthy skin available; however its exact usefulness should be carefully recognised [14, 15]. 

**Figure 2:**
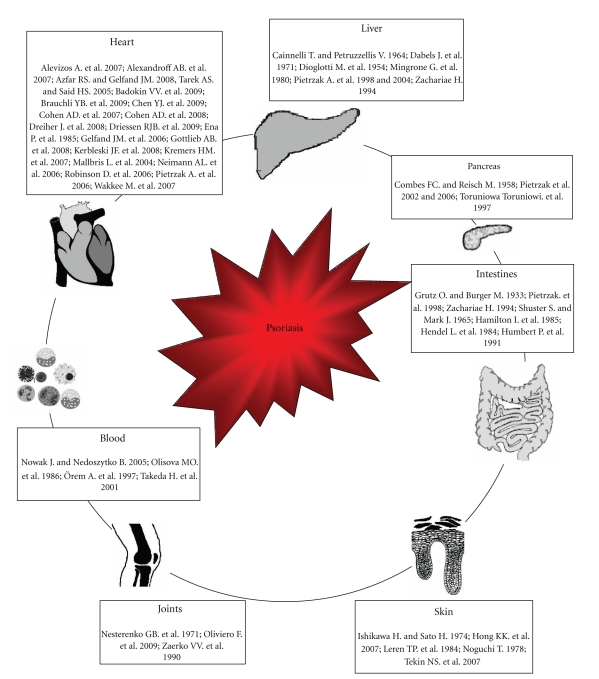
Influence of the psoriasis associated dyslipidemia on human organs. This figure is based (after permission) on the figure from [19].

Nowadays the studies are concentrated on the skin surface lipids, epidermal lipids (including stratum corneum lipids, and epidermal phospholipids), serum lipids, dermal low-density lipoproteins in the psoriatic skin, lipid metabolism, oxidative stress and correlations between inflammatory parameters, lipid parameters, and clinical symptoms of the disease (Figure 2) [10–12, 16–19]. The aim of this study is to present an update of the lipid studies in psoriasis on the basis of the literature review.

## 2. Skin Surface and Epidermal Lipids

The stratum corneum consists of corneocytes and intracellular lipids, mainly ceramides, sterols, and free fatty acids which form the barrier for diffusion of substances into the skin [20–23]. The lipids are organised into multilamellar intercellular membranes derived from glycerophospholipids, glucocerebrosides, sphingomyelin of the stratum granulosum-stratum corneum interface [23, 24]. Then the precursors are converted to ceramides and free fatty acids by the hydrolytic enzymes [25, 26]. In psoriasis, alterations in ceramide content have been demonstrated [27] and abnormal lipid structures reported [28]. Total lipids, phospholipids, triacylglycerols, and cholesterol were found to increase both in blood and in epidermis of psoriatic patients [29, 30]. The proportion of an esterified fraction decreased mainly in the normally appearing epidermis areas, especially in severe psoriasis [31]. In the gas liquid chromatography, significantly lower spectrum of short-chain fatty acids (SCFAs) levels were detected in both psoriatic and uninvolved areas [32]. The correlation was found between increased levels of free and total cholesterol as well as phospholipids in the epidermis and the severity of psoriasis [31, 32]. 

The main features of the corneous layer observed under the scanning electron microscope include widened intracellular spaces, lack of resistant intercellular junctions, impaired intracellular adhesion, which may result in markedly abnormal cholesterol homeostasis [33, 34]. In the lipid thin-layer chromatography, an increased amount of total phospholipids was found in the involved psoriatic epidermis whereas the decrease of phosphatidylserine and the increase of phosphatidylinositol were observed in psoriatic lesions and in the lesion-free epidermis [35]. 

Lacroix demonstrated significant amount of cholesterol in scaly plagues and in serum. He suggested that psoriasis might be the form of cholesterol elimination through the skin [9]. The regulation of cellular cholesterol metabolism is already fully developed in the foetal life. The maintenance of its steady cellular levels is an important element of cellular and systemic homeostasis. It is already known that this homeostasis is disturbed in psoriasis [10]. Every day about 85 mg of cholesterol is secreted through the healthy skin. In psoriasis, the patients lost daily 12–23.5-fold more lipids with the scales than healthy subjects [18, 36, 37]. 

## 3. Serum Lipids

Serum lipids levels were examined in many different groups of psoriatic patients in comparison to relevant healthy controls [9–11, 16, 18, 38–48]. The blood lipid results are considerably dependent on group matching (age, gender, and ethnic and cultural factors). In most of the studies, a statistically significant elevated level of total cholesterol (TC), low-density lipoprotein (LDL) cholesterol and/or triglycerides (TG) in psoriatic patients was demonstrated comparing to a healthy control group [11, 16, 18, 39, 40, 43–47, 49–52]. Moreover, there was a decrease of high density lipoprotein (HDL) cholesterol in the serum of psoriatic patients [43, 48, 50–53]. Only in a few studies no differences in lipid serum levels between psoriatic patients and healthy controls were observed [38, 42, 54]. 

**Figure 3:**
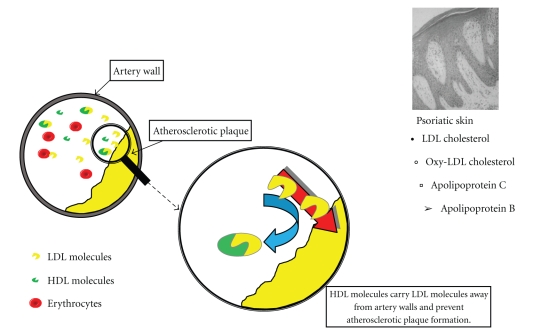
Lipoprotein replacement in circulation from artery walls and peripheral blood into psoriatic skin lesions. Based and modified with permission from figures from [56]. Available from http://www.biolsci.org/v05p0474.htm.

Nowadays there is an increased interest in HDL cholesterol, because clinical and epidemiological studies showed an inverse relationship between the level of HDL and the development of atherosclerosis [55]. HDL is a very important factor in reverse cholesterol transport (RCT). It takes part in the transport of cholesterol produced or accumulated in the peripheral tissues to the liver or other steroidogenic tissues and exerts the antioxidant, anti-inflammatory, antithrombotic and fibrinolytic action [55]. It should be underlined that neither HDL nor LDL is “bad cholesterol,” because both are essential for the proper transport of cholesterol (Figure 3).

Results of the studies present a decrease of cholesterol and phospholipids levels connected with HDL fraction independently of psoriasis severity and duration [36]. In psoriasis, a decrease of HDL synthesis and HDL structural changes can be observed, due to various biochemical disturbances, such as abnormalities of receptor function, changes of hepatic structure and function, activity changes of hepatocyte membranes, impaired RCT, esterification, and lipases [36]. It can be hypothesised that HDL structural changes are caused by a decrease of cholesterol and phospholipids level as well as an increase of apolipoprotein A (apoA) concentration in the HDL coat. So far, all the studies were based on the quantitative evaluation of lipids in the psoriatic patient serum and epidermis. Further studies are needed to specify the role of disturbances of HDL structure and composition as well as connections between lipid abnormalities and the immune response in psoriasis. 

The studies concerning the concentration of serum phospholipids in the psoriatic patients present different results. A decrease of concentration of total phospholipids, as well as phosphatidylethanolamine, lecithin, the lecithin : cholesterol ratio and linolenic acid, docosatetraenoic acid, docosapentaenoic acid, and docosahexaenoic acid in the serum was observed [57–61]. There was also an increased level of some fractions of serum phospholipids (e.g., lysolecithin) and palmitic acid, palmitoleic acid, and dihomo-*γ*-linolenic acid (DHLA) [57, 58, 62–64]. Some reports, however, do not present any differences in the level of serum phospholipids between psoriatic patients and healthy control group [65]. Our results did not show any statistically significant differences in the level of total phospholipids, but the decreasing tendency of its level was seen in both normolipidemic and hyperlipidemic patients [10].

## 4. Apolipoproteins

Apolipoproteins are the protein part of lipoproteins, and their composition is specific for each lipoprotein. They have a different molecular structure, amino acid composition, and antiatherosclerotic properties. In psoriatic patients, different results concerning apolipoproteins apoA1, apoB, and apoE were presented [16, 41, 66, 67]. Apolipoprotein A1 has been immunocytochemically detected at the psoriatic skin dermoepidermal junction, vascular walls, and the perivascular region of papillary dermis. Apolipoprotein B100 and apolipoprotein E were observed intracellularly both in normal epidermis and psoriatic epidermis, and they were also detected in parakeratotic regions in the horny layer [68]. 

ApoA1 plays the main part in the reverse cholesterol transport from the peripheral cells to the liver. Its decreased level has an influence on the higher risk of atherosclerosis development [69]. ApoA2 stabilizes the HDL structure and is considered as the lecithin : cholesterol acetyltransferase (LCAT) inhibitor. Its role concerning atherosclerosis is controversial, because it was shown that apoA1 impaired the inflow of cholesterol from adipocytes to the extracellular space [70]. Elevated levels of apolipoproteins A1 and A2 accompany the intake of alcohol. The level of apoA1 increases also in familiar hyperproteinemia, in pregnancy, during estrogen therapy, and during physical exercise. 

Elevated levels of apolipoprotein B are associated with the increased risk of atherosclerosis, due to its role in the cholesterol accumulation in the endothelium, which initiates the atheromatous process. Apo B elevated levels are observed in the hyperlipidemia type IIa, IIb, IV, and V, in nephritic syndrome, pregnancy, familiar hyperapo-ß-lipoproteinemia, biliary obstruction, smokers, and dialyzed patients on treatment with diuretics ß-blockers, cyclosporine, or glucocorticoids [71].

Apolipoprotein C3 (apoC3) is suggested to inhibit lipoprotein lipase [72, 73] and hepatic triglyceride lipase [74], enzymes responsible for the clearance of triglyceride rich particles from the plasma. Furthermore, apoC3 was shown to inhibit the hepatic uptake of triglyceride rich particles [75]. Apo C3 also appears to interfere with HDL receptor-mediated uptake of lipoproteins. It is known that an increase in apoC3 levels induces the development of hypertriglyceridemia.

In most studies, elevated levels of apoA1, apoB [16, 43], apoC3, and apoE [41, 76–78] were detected in the serum of psoriatic patients compared to the healthy control group. However, there are also contrary results showing decreased levels of apolipoproteins [79]. Many authors did not show any differences in apoA1, apoA2, and apoB levels between psoriatic patients and the control group [10, 76, 80]. It was also reported that apoA1 sequestration in the inflamed tissues might lead to reduced HDL-C serum levels and thus increase the risk of cardiovascular disease in psoriatic patients [81]. 

Apolipoprotein E (ApoE) is a glycoprotein involved in the regulation of triglycerides and low-density lipoprotein (LDL) levels [67]. ApoE can modulate mitogen-activated T-lymphocyte proliferation in vitro and provides protection against some infections [82, 83]. The role of the apoE gene in psoriasis was suggested, because in psoriatic skin there is the downregulation of ApoE expression and the normalization of ApoE levels precedes clinical improvement [67]. Furthermore, in a Japanese population the epsilon 2 allele was found to be significantly more frequent in psoriatic patients than in controls, suggesting that there may be a relationship between these particular alleles and development of psoriasis [84]. It is believed that the epsilon 4 allele could be a risk factor for developing a severe form of psoriasis [85]. 

## 5. Oxidative Stress

Reactive oxygen species (ROSs) such as hydroxyl radical (HO^*∙*^), peroxyl radicals (ROO^*∙*^), superoxide anion (O_2_^∙−^),hydrogen peroxide (H_2_O_2_), nitrogen oxide (NO^*∙*^), and hypochlorous acid (HOCl) are constantly produced as a result of metabolic reactions in living systems [86]. Oxidative stress may be defined as an imbalance between cellular production of ROS and antioxidant defence mechanisms. It leads to oxidative damage of lipids and proteins contributing to barrier integrity, which is essential for healthy skin conditions [18, 87, 88]. The skin antioxidant system consists of a network of both enzymatic (glutathione peroxidase (GSH-Px), catalase (CAT), superoxide dismutase (SOD), and paraoxonase (PON1)) and nonenzymatic antioxidants. Nonenzymatic antioxidants (glutathione, *β*-carotene, ascorbic acid, and tocopherols) present in cells are regarded as protectors against the lipid peroxidation [88]. 

Increased production of oxygen metabolites, overwhelming the antioxidant capacity of the body, is an important feature in psoriasis [87]. Early and active psoriatic lesions are characterized by the intraepidermal penetration of activated polymorphonuclear leucocytes which leads to *ROS* production provided by *NADPH* oxidase and proteolytic enzymes [88]. The production of ROS can be indirectly assessed by the levels of lipid peroxidation products such as lipid hydroperoxide (LHP), malondialdehyde (MDA), oxidized low-density lipoprotein (ox-LDL), and thiobarbituric acid (TBA) [87]. Patients with psoriasis exhibit increased concentrations of MDA [51, 87, 89, 90] and ox-LDL [18] in the tissues and higher levels of TBA [43, 52, 87] and anti-ox-LDL autoantibody (AuAb-oxLDL) [50, 51, 87] in the blood. The lipid peroxidation markers were found significantly higher in the patients with severe or active psoriasis (PASI > 3) than in the patients with mild or inactive psoriasis (PASI < 3) [43]. The accumulation of ox-LDL was detected in the upper epidermis of the involved skin from psoriatic patients by direct immune-fluorescent method [18]. Ox-LDLs are able to initiate inflammation and to influence the adhesion of endothelial cells and on oxidant status of the blood vessels cells, which is important in the development of early atherogenesis [53]. They are also antigenic and can elicit an immune response with a generation of circulating antibodies AuAb-oxLDL and *β*2-GP1-dependent anticardiolipin antibodies (aCL), as a consequence of structural similarity between ox-LDL surface structure and *β*2-GP1-anionic phospholipid complex, the antigenic target for aCL [91]. The level of AuAb-oxLDL has been suggested to reflect the in vivo oxidation of LDL. The importance of AuAb-oxLDL in diseases such as myocardial infarct, atherosclerosis, diabetes mellitus, renal failure, systemic lupus erythematosus (SLE), rheumatoid arthritis (RA), Beh*ς*et's disease, and psoriasis was suggested [51]. aCL level is increased in psoriatic patients. It could be a useful marker in predicting atherosclerosis risk, because it may promote atherosclerotic lesions [91]. In plasma and red blood cells (RBCs) of psoriatic patients, increased levels of MDA were observed which indicates an advanced peroxidative process in erythrocyte membranes. The increased peroxidation of lipid bilayer caused by a decrease of antioxidant enzyme activities may be the essential mechanism of the membrane fluidity decrease observed in association with the exacerbation of the disease [88, 89, 92]. The impaired antioxidant status is shown by decreased serum levels of erythrocyte SOD [51, 90] and GSH-Px activities [51, 90, 92, 93] of increased PON1 activity [54] and of increased [90] or decreased [51] serum CAT activity in patients with psoriasis. Nonenzymatic antioxidants were also decreased [51, 92, 93]. Changes in the elastase neutrophil ratio illustrating an increase in neutrophil function can be a marker of psoriasis [43]. In general, total antioxidant status (TAS) in psoriasis is reduced [43, 51], or there are no significant differences between patients and healthy controls [52, 54, 89]. 

A high serum total homocysteine (tHcy) level was observed in patients with psoriasis. The main mechanisms of hyperhomocysteinemia engaged in the development of atherothrombosis are endothelial injury, platelet activation, oxidative modification of low-density lipoproteins, and endothelial-leukocyte interactions [94, 95]. There was a positive relationship between an increased level of AuAb-oxLDL and plasma tHcy levels which may play an important role in development of atherothrombotic complications in psoriatic patients [96].

Oxidative stress may have a pivotal role in both therapeutic mechanisms and side effects induced by anthralin. Systemic antioxidant administration may provide an opportunity for therapeutic intervention against anthralin-associated toxicities [88]. Lipid peroxidation is the earliest response mediating activation of downstream signalling events in peripheral blood mononuclear cells (PBMCs) and keratinocytes by anthralin. It leads to the activation of c-jun-N-terminal kinase (JNK), event relevant for the regulation of cellular proliferation and apoptosis [97].

It is well known that phototherapy is recommended in the psoriasis treatment. However, both ultraviolet A and B radiation (UVA and UVB) apart from therapeutic and immunomodulating action induce production of ROS and increase lipid peroxidation [53]. There was a difference between the effect of phototherapy on lipid parameters in patients with mild or moderate psoriasis (PASI1 from 5.4 to 22.1; mean 15.2 ± 4.9) and severe psoriasis (PASI 2, PASI 22.5 to 49.2; mean 30.3 ± 5.8). Exacerbated skin manifestations of psoriasis are accompanied by an increase of dyslipidaemia and oxidation processes. Therefore patients with severe psoriasis are exposed to higher risk of atherosclerosis. PASI2 patients have higher level of AuAb-oxLDL than PASI1 patients. Phototherapy increased TC, LDL, and AuAb-oxLDL level in PASI1 patients. Level of ox-LDL was decreased after phototherapy in patients with severe psoriasis and it was accompanied by increase of ferric reducing ability of plasma (FRAP) and negative correlation with AuAb-oxLDL level. It can be explained by therapeutic action of phototherapy and reduction of inflammatory processes [53]. 

## 6. Peroxisome Proliferator-Activated Receptors (PPARs) and Liver X Receptors (LXRs)

The epidermis is a very active site of lipid metabolism, and all peroxisome proliferator-activated receptor (PPAR) and liver X receptor (LXR) isoforms are expressed in the epidermis. An increased expression of PPAR*β*/*δ* and a decreased expression of PPAR*α* and PPAR*γ* were observed in the lesional skin of patients with psoriasis and atopic dermatitis [98–100]. Since the prevalence of metabolic syndrome is increased in psoriasis [101], a combination of insulin resistance, obesity, or chronic inflammation may trigger the expression of PPAR*β*/*δ*, which in turn contributes to a nonterminated regenerative skin phenotype. This disease mechanism would be expected to be aggravated by acute inflammation, or stress via the induction of PPAR*β*/*δ* by TNF*α* and stress-activated kinase [102]. 

PPARs *α*,  *β*/*δ*, *γ*, and LXRs *α* and *β* belong to the nuclear steroid hormone receptor superfamily, which are regulated by fatty acid derivatives capable of controlling lipid and lipoprotein metabolism, cell proliferation, differentiation, and apoptosis of various cell types, including keratinocytes and sebaceous gland cells. These receptors play also a role in cutaneous carcinogenesis [100]. 

An activation of PPARs and LXRs leads to stimulation of epidermal lipid synthesis, formation and secretion of lamellar bodies, and activation of enzymes required for the extracellular processing of lipids in the stratum corneum, resulting in the formation of lamellar membranes that mediate permeability barrier function. PPAR*γ* activation appeared to have the least effect on epidermal lipid synthesis among the PPAR and LXR activators tested. PPAR*β*/*δ* is the key PPAR isoform involved in lamellar body formation and secretion as well as in lipid storage [103, 104]. 

PPAR-*α* can also modulate the inflammatory response by inhibiting cytokine secretion, maturation, and migration and the T-cell-stimulatory activity of the epidermal antigen-presenting cell, the Langerhans cell. This was associated with decreased levels of phosphorylated nuclear factor-*κ*B (NF-*κ*B) [105]. Moreover, PPAR-*α* activation induces antioxidant enzymes, such as catalase or SOD, which would reduce the oxidative stress and the activation of mediators of inflammatory response [88]. The anti-inflammatory role of PPAR*β*/*δ* and PPAR*γ* in the skin is uncertain, but it is suggested that they downregulate inflammation. LXR activators have a potent anti-inflammatory activity in both the irritant and allergic contact models of cutaneous inflammation [106, 107]. These findings suggest the possibility of PPAR-*α* activators as novel nonsteroidal anti-inflammatory drugs in the topical treatment of common inflammatory diseases such as atopic dermatitis, psoriasis, acne, and photodermatitis. A great improvement of skin lesions and also of psoriatic arthritis had been initially documented in patients with psoriasis treated with the oral PPAR*γ* activators troglitazone [108, 109] or pioglitazone [110–112]. In contrast, topical treatment of psoriatic skin with the PPAR activators tetradecylthioacetic acid and rosiglitazone did not show a significant effect [113, 114]. 

LXR and PPAR influence also the synthesis of cholesterol sulfate, which is a potent regulator of epidermal differentiation and corneocyte desquamation. The stimulation of both the cellular and extracellular components of the stratum corneum by PPAR*α* and LXR activators results in the generation of a mature, functionally competent stratum corneum earlier in fetal development. Moreover, in a mouse model of epidermal hyperproliferation induced by repeated barrier disruption to the flank skin of hairless mice [115], topical PPAR*α* activation inhibited proliferation and increased keratinocyte apoptosis. The activation of PPAR*α* in the epidermis decreases keratinocyte proliferation. The absence of PPAR*β*/*δ* leads to increased keratinocyte proliferation and under some experimental conditions PPAR*β*/*δ* activators inhibit keratinocyte proliferation. It has been demonstrated that activation of PPAR*β*/*δ* induces endothelial cell proliferation and angiogenesis [116]. It was suggested that in the hyperproliferative epidermis of psoriatic skin, PPAR*β*/*δ* overexpression mediates keratinocyte proliferation via NF-*κ*B [98]. The proliferative state of the keratinocytes may determine the effect of PPAR*γ* activation on keratinocyte proliferation. A proapoptotic effect of PPAR*γ* in T cells has been observed [117], and activation of PPAR*γ* has an inhibitory effect on psoriasis, whereas this is not the case with PPAR*β*/*δ* activation [118, 119]. In LXRs deficient mice, thinning of the epidermis was observed [120]. 

## 7. Cardiovascular Disease (CVD)

In patients with psoriasis one observes an increased risk of cardiovascular disease which can be explained by several possible biological factors [6, 121–125]. Psoriasis is associated with traditional risk factors of CVD such as increased BMI, hyperlipidemia, hypertension, type 2 diabetes mellitus, and cigarette smoking [124–126]. Obesity has been shown to be an independent risk factor for the development of psoriasis and is also associated with more severe psoriasis and cardiovascular complications [125]. The persistent skin inflammation may contribute to a dyslipidemia and premature atherosclerosis [126, 127]. The duration of disease and its severity are related to the incidence of cardiovascular diseases, such as myocardial infarction, coronary artery disease and stroke [16, 38–40, 43–47, 54, 101, 121, 122, 127–135]. In psoriatic patients, lipid abnormalities are correlated with increased mortality due to myocardial infarction and stroke [129, 134]. Elevated level of C-reactive protein (CRP) is a risk factor for CVD and it can predict long-term risk for cardiovascular events [136]. The treatment for psoriasis such as retinoids and cyclosporine may be also responsible for initiation of hyperlipidemia which can promote future CVD [137–141]. Methotrexate use is associated with hyperhomocysteinemia, also a risk factor for cardiovascular disease [142].

**Figure 4:**
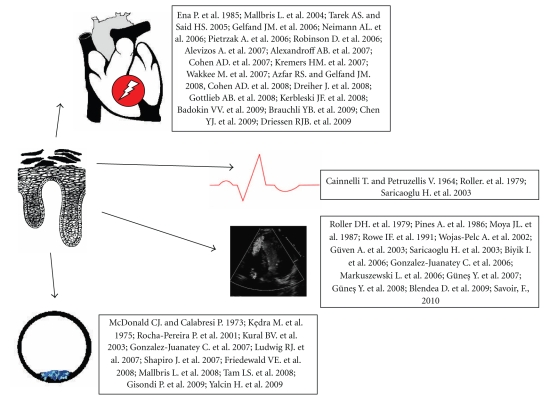
Psoriasis and cardiovascular abnormalities. This figure is a modified one (after permission) from [19].

There was a strong association observed between arterial stiffness, which is endothelial dysfunction marker, and the risk of cardiovascular events. Pulse wave velocity (PWV) is the gold standard measurement of arterial stiffness and in the patients with psoriasis and psoriatic arthritis an increased femoral-carotid PWV was observed [127, 143]. There were also functional and structural changes in the myocardium, changes in electrocardiographic activity, such as increased P wave dispersion, and structural changes in coronary vessels in psoriatic patients (Figure 4) [7, 19, 144]. 

## 8. NTproBNP

In recent years, the probable usefulness of NTproBNP as a biomarker of heart failure (HF) has been established. There was a positive correlation observed between NT-pro BNP in blood serum of psoriatic patients and heart diseases as well as acceptation of the disease [145]. 

## 9. Lipid and Immunologic Abnormalities

In psoriasis, the association between lipid and immunologic abnormalities was observed, that is why the disease could be described as an immunometabolic syndrome [128, 146]. Psoriasis is a chronic inflammation characterized by increased Th-1 and Th-17 T cell activity [128]. The significant role of cytokines, such as TNF-*α*, IL-6, IL-8, IFN-gamma, IL-1, and IL-17 in the generation of proatheromatous abnormalities (dyslipidemia, insulin resistance, endothelial dysfunction, clotting system activation, and pro-oxidative stress) was reported [127, 128, 146, 147]. TNF-*α* is a potent activator of c-Jun amino-terminal kinase,which stimulates the main regulator of proinflammatory activity protein-1 and is connected with obesity [128]. TNF-*α* can also lead to insulin resistance by inhibiting phosphorylation of insulin receptor tyrosine and of insulin receptor substrate 1. Treatment with TNF-*α* inhibitors affects the increase of HDL level [128]; in particular, TNF may affect endothelium dysfunction by decreasing the levels of nitric oxide synthase and cyclooxygenase 1 [127].

## 10. Effects of Antipsoriatics and Hypolipemic Drugs on Psoriasis

Antipsoriatic drugs can be also responsible for the lipid profile disturbances in psoriatic patients, because of their action on the circulating lipids [148–156]. Retinoids have the most potent activity on increasing the levels of triglycerides, total cholesterol, LDL cholesterol, and VLDL cholesterol and simultaneously decreasing the HDL fraction [137–140]. There are some reports that the diet enriched with fish oil can reduce side effects of these drugs [157, 158]. Cyclosporin has milder effects on the lipid profile, but it can also lead to some abnormalities for example TG elevation [159]. TNF-*α* inhibitors can cause an increase of serum triglyceride levels, but they have beneficial effects on the increase of HDL level and are able to decrease blood insulin levels [141, 160–162]. 

Hyperlipidemia is treated with statins which effectively reduce CRP and TNF-*α* levels as well as decrease levels of low-density lipoproteins and alleviate the arterial stiffness. Statins also downregulate adhesion molecules such as LFA-1 and ICAM-1 on leukocytes and endothelial cells which are essential in leukocyte activation, leukocyte migration to inflammatory sites, and immunologic cytotoxicity [163]. Statins have the inhibiting action on the expression of MHC II molecules, chemokine receptors on Th-1 cell and the production of NO [163]. These drugs are generally beneficial for psoriatic patients and reduce the risk of cardiovascular diseases. However, there was also a case of exacerbation of psoriasis after the treatment with three different statins and bezafibrate [164]. Fibrates, used to decrease cholesterol levels, may also affect rapid and acute development of clinical symptoms of psoriasis. 

## 11. Summary

The lipid disturbances are recognised as a very important part in the pathogenesis of psoriasis. The results of the majority of the studies are coherent and indicate that the increased total cholesterol, LDL cholesterol and/or triglycerides, and decreased HDL cholesterol in psoriatic patients' serum the composition of apolipoproteins, and increased production of oxygen metabolites are features of the metabolic syndrome. These factors have also a great impact on some comorbidities observed in psoriatic patients especially on cardiovascular diseases. These lipid disturbances are also connected with immunological abnormalities, that is why psoriasis could be classified as an immunometabolic disease. In spite of the intensive investigations, the explanation of the steps of disease mechanisms in psoriasis have not been recognised so far. On the basis the literature data, further studies should be designed to connect the lipid and immunological disturbances. 

The review of the last years suggests an introduction of some new therapeutic methods for psoriatic patients as for example statins. Their immunomodulatory activities like influence on T cells and antigen presenting cells function, influence on leukocyte adhesion and endothelial cell function are discussed. In many papers the importance of reduction of animal fat, introduction of fish and plant oil, preparations with the omega-6 and omega-3 fatty acids as well as BMI reduction, prevention of obesity and quitting addictions were suggested.
